# Correction: Deep fusion of gray level co-occurrence matrices for lung nodule classification

**DOI:** 10.1371/journal.pone.0324959

**Published:** 2025-05-20

**Authors:** The PLOS One Editors

The articles cited in [1] as References 13, 15, and 52 were retracted after [1] was published due to concerns about potential manipulation of the publication process.

In addition, the LIDC-IDRI dataset citation [2] is missing from the ‘Dataset’ section and References list, and the Data Availability statement is incomplete and includes a link that is not currently functional. The Data Availability statement is updated to:

This study uses data obtained from the LUNGx and LIDC-IDRI datasets. The LIDC-IDRI dataset is available at https://www.cancerimagingarchive.net/collection/lidc-idri/. The LUNGx dataset is freely available from the following website: https://wiki.cancerimagingarchive.net/display/Public/LUNGx+SPIE-AAPM-NCI+Lung+Nodule+Classification+Challenge.
